# The Semantic Processing of Motion Verbs: Coercion or Underspecification?

**DOI:** 10.1007/s10936-016-9466-7

**Published:** 2016-12-09

**Authors:** Julia Lukassek, Anna Prysłopska, Robin Hörnig, Claudia Maienborn

**Affiliations:** 1 German Department, Universität Tübingen, Wilhelmstraße 50, Tübingen, Germany; 2 SFB 833, Universität Tübingen, Nauklerstraße 35, Tübingen, Germany

**Keywords:** Motion verbs, Aspectual coercion, Underspecification, Semantic processing

## Abstract

Underspecification and coercion are two prominent interpretive mechanisms to account for meaning variability beyond compositionality. While there is plentiful evidence that natural language meaning constitution exploits both mechanisms, it is an open issue whether a concrete phenomenon of meaning variability is an instance of underspecification or coercion. This paper argues that this theoretical dispute can be settled experimentally. The test case are standard motion verbs (e.g. *walk*, *ride*) in combination with ±telic directional phrases, for which both underspecifaction and coercion analyses have been proposed in the literature. A self-paced reading study which incorporates motion verbs, directional phrases and durative/completive temporal adverbials (1) aims at determining the aspectual value of such verbs, and (2) compares the hypotheses of the Underspecification and Coercion Accounts. The results of the reading time experiment (flanked by a corpus study and a completion study) indicate that motion verbs are aspectually underspecified. They combine with ±telic directional phrases with equal ease. The combination with a mismatching temporal adverbial is an instance of coercion, causing additional processing costs.

## Introduction

Coercion and underspecification are two prominent mechanisms of meaning adaptation. Coercion provides a contextually licensed repair of a combinatorial conflict and hence standardly involves a grammatically ill-formed structure (cf. Asher 2011; Pustejovsky 2011). Underspecification relates to a contextually driven specification of a grammatically well-formed, yet underspecified structure (cf. Bierwisch 1982, 1983; Egg 2005). Although the existence of these mechanisms is widely agreed upon, controversy remains whether particular phenomena are best modeled as instances of one or the other. The present paper explores these phenomena in a new empirical domain, focusing on motion verbs as an experimental test case.

Motion verbs like *sail*, *run* and *walk* can head telic or atelic verbal phrases (VPs). This can be demonstrated by combining them with different types of directional phrases, e.g. in (1). These directional complements play a crucial role in the determination of the aspectual properties of a VP headed by a motion verb (see Bierwisch 1988; Maienborn 1990 for a discussion of the complement status of directional phrases in combination with motion verbs).

For instance, in (1a) and (1c) the German motion verb *segeln* ‘to sail’ combines with an atelic directional phrase such as *entlang der Küste* ‘along the coast’. This combination yields an atelic VP (marked below as [−telic]).^1^ Atelic VPs combine smoothly with durative temporal adverbials such as *drei Tage lang* ‘for three days’ in (1a). But they yield an aspectual mismatch in combination with a completive temporal adverbial such as *in drei Tagen* ‘in three days’ as in (1c). The same motion verb can also appear in a VP with a telic directional phrase such as *zur Schatzinsel* ‘to the treasure island’ in (1b) and (1d). In this case, the overall VP is telic as well (marked below as [$+ telic$]). Telic VPs show the opposite behavior with respect to durative versus completive temporal adverbials; see (1b) versus (1d). 
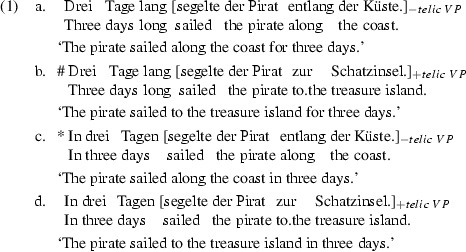


Under certain circumstances, an aspectually specified VP can have its aspectuality changed by temporal adverbials such as *for X time* or *in X time*. This shift from one aspectual value to another is widely discussed in the literature on the phenomenon of so-called “aspectual coercion”; see, e.g. Moens and Steedman (1988), Rothstein (2004), van Lambalgen and Hamm (2005), De Swart (2011), Arsenijević et al. (2013), Dölling (2014).

The temporal adverbials target VPs with a specific aspectual type and they have the ability to adjust their target to this specific type, if it does not have the required properties. Let us illustrate this on the sentences in (1). As indicated above, the aspectual values of the temporal adverbials and the VPs match in examples (1a) and (1d): In (1a), the durative temporal adverbial is compatible with the atelic VP, and in (1d), the completive temporal adverbial is compatible with the telic VP. However, there is an aspectual mismatch between the temporal adverbials and VPs in (1b) and (1c): In (1b), the telic VP doesn’t match the durative temporal adverbial and in (1c), the atelic VP doesn’t match the completive temporal adverbial. In (1b), the aspectual conflict could be resolved by imposing an atelic reading onto the VP, in this case most plausibly an iterative one (# signals a semantic anomaly due to type mismatch) — the pirate sailed to the treasure island and back over the course of three days. But not all aspectual mismatches can be resolved: there is no straightforward repair of a combinatory conflict for sentence (1c) (* signals ungrammaticality).

Thus far we have considered cases where the VP appears to inherit the aspectual value of the directional phrase, i.e. where the verb has no visible impact on the aspectual interpretation of the VP. This may give the impression that the verb is not lexically specified with respect to telicity. Nevertheless, there are examples of motion verbs which carry specific aspectual information, like *streunen* ‘to roam’ in (2). 
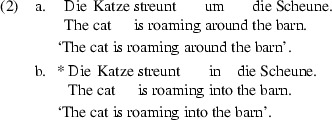


The German motion verb *streunen* ‘to roam’ is compatible with an atelic directional phrase such as *um die Scheune* ‘around the barn’ in (2a), but is incompatible with the telic directional phrase *in die Scheune* ‘into the barn’ in (2b). Note that this aspectual mismatch cannot be resolved. Sentence (2b) is plainly ungrammatical. This example highlights that there are motion verbs which have a lexically specified aspectual value [e.g. (2)], while others appear to be neutral in this respect (e.g. [1)]. This raises new questions as to the aspectual value of standard motion verbs such as *run*, *sail*, *walk*. How does the composition of a VP headed by such motion verbs proceed? Two possibilities come to one’s mind.

First, typical motion verbs like *run*, *walk*, *sail* can be considered underspecified w.r.t. telicity. If underspecified, these verbs are expected to combine equally well with both telic and atelic directional prepositional phrases because they can take on the aspectual value of the directional phrase; cf. Maienborn (1990). Under this assumption, standard motion verbs introduce a dynamic eventuality, but they do not specify its telicity value. The composition of a VP headed by an underspecified motion verb is straightforward: the underspecified meaning representation is compositionally specified when needed (for instance when combined with a telic or atelic directional phrase) or it may remain underspecified (cf. Bierwisch 1982, 1983; Egg 2005; Dölling 2014).

Consider again the sentences in (1). Since the motion verb is underspecified w.r.t. telicity, the aspectual value of the VP is specified by the directional phrase. In (1a) and (1d) the aspectually specified VP and the temporal adverbial match. The aspectual conflicts in (1b) and (1c) arise due to the aspectual mismatch between the temporal adverbial and the VP, which inherits its aspect from the directional phrase.

Alternatively, standard motion verbs may be considered as being lexically specified to the atelic aspect (see, e.g. Dowty 1979; Moens and Steedman 1988; Rothstein 2004; Bott 2010). In fact, motion verbs are often mentioned as prototypical exemplars of activity expressions. If this is the case and motion verbs are lexically specified with respect to telicity, then they should contribute to the aspectual value of a sentence. Under this assumption, a verb like *segeln* ‘to sail’ in (1) is a lexical activity expression that carries a $[-telic]$ feature. The aspectual value of the verb and the directional phrase in (1a) and (1c) match, and hence the meaning of the VP is derived compositionally [the same is true of sentence (2a)].

In (1b) and (1d), in contrast, there is an aspectual conflict within the VP between the verb and the directional phrase. This VP-internal conflict is resolved by coercion (Moens and Steedman 1988) in both (1b) and (1d): the telic directional phrase coerces the atelic motion verb to a telic interpretation, whereby the overall aspectual value of the VP is $[+telic]$. There may be more than one aspectual conflict within a sentence, as it is the case in (1b). Here, the aspectual value of the VP is $[+telic]$ and it clashes with the durative temporal adverbial, necessitating another repair via aspectual coercion. However, the aspectual conflict can not always be resolved, as in the case of (1c).

Underspecification and coercion are extensively discussed in the current literature. It is intriguing, though, that the proposed formalisms tend to collapse these two kinds of meaning adaption into either underspecification-based or coercion-based accounts.^2^ Our test case of motion verbs is well-suited to compare the predictions of an underspecification approach and a coercion approach experimentally, because they differ relative to the number and location of assumed aspectual mismatches and their resolution via aspectual coercion. A lexical underspecification account posits aspectual mismatches only between aspectually specified VPs and temporal adverbials as in (1b) and (1c). A lexically grounded coercion account assumes that motion verbs are specified relative to their telicity, and therefore assumes that there is an additional VP-internal aspectual conflict between the motion verb and its directional complement in the case of (1b) and (1d). We will call the two approaches *Underspecification Account* and *Coercion Account*, respectively. The aim of the present paper is to decide upon the two accounts to the contribution of standard motion verbs to the aspectual value of a VP by testing their predictions on aspectual coercion. Thereby we aim to show that the theoretical dispute whether a given phenomenon of meaning adaption is an instance of underspecification or coercion can be resolved experimentally.

Aspectual coercion has been the subject of several psycholinguistic studies (see, e.g. Piñango et al. 1999, 2006; Todorova et al. 2000; Seegmiller et al. 2004; Husband 2006; Pickering et al. 2006; Brennan and Pylkkänen 2008; Townsend 2013). These studies have shown that aspectual coercion is a process which leads to increased cognitive effort. Piñango et al. (1999) investigated sentence pairs like the one in (3) in a sentence-comprehension experiment with a secondary lexical decision task. They reported longer reaction times for (3a) in comparison to (3b) and interpreted this finding as evidence for costly aspectual coercion of the VP triggered by the temporal adverbial; as a result of coercion, (3a) has an iterative meaning. 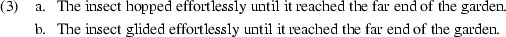


Todorova et al. (2000) investigated similar sentences with iterative readings, like (4), in a reading-time and stop-making-sense experiment. They reported longer reading times for (4a) in comparison to (4b). Todorova and colleagues interpreted this finding as evidence for the costliness of the aspectual coercion between the VP and the temporal adverbial, in contrast to cases where the iterative reading is achieved by compositional means, e.g. plural *checks* in (4b). 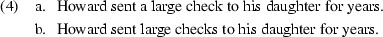


Pickering et al. (2006) reported a series of experiments in which they replicated the experiments of Piñango et al. (1999) and Todorova et al. (2000) with slight changes. Unlike the earlier studies, Pickering and colleagues reported no processing difficulties when a telic VP mismatches a durative adverbial phrase. They argued that, while it is true that aspectual coercion is tied to processing difficulties, the meaning representation of a VP headed by verbs such as *hop* and *send* (a cheque) is underspecified w.r.t. telicity, because both a telic (singular) and atelic (iterative) reading is equally plausible. The comprehenders commit to an interpretation only when the intended interpretation is clear.

To date, the focus of most studies on aspectual coercion lies on the case of iterative coercion. Bott (2010) is the first to systematically differentiate different types of aspectual coercion. Most importantly for our present purposes, the results of his experiments indicate that additive coercion, that is, the addition, for instance, of a culmination point to an activity, is taxing. The kind of aspectual conflict that the Coercion Account assumes for (1b)/(1d) belongs to this class of additive coercions: A goal is added to an unbounded movement turning an atelic activity verb into a telic VP. Against this background, we take the existing evidence to corroborate processing costs associated with the kinds of aspectual coercion that are relevant for the assessment of (1).

## Rationale of the Study and Predictions

We designed a self-paced reading study which incorporates aspectually sensitive directional phrases and temporal adverbials (as do Piñango et al. 1999, 2006; Todorova et al. 2000; Pickering et al. 2006), as well as standard motion verbs, i.e. motion verbs such as *walk*, *run*, *sail*, *ride* etc. (but not *roam*) that appear to combine smoothly with both telic and atelic directional phrases. The study aims at (1) determining the aspectual value of standard motion verbs; and (2) providing evidence that helps to decide the theoretical dispute about the adequate modeling of the aspectual interpretation of VPs headed by motion verbs. Our design has the advantage over previous studies in that the predictions of the two accounts correspond to different interaction patterns instead of the mere presence or absence of coercion effects.

Example (5) shows the four variants of a sample stimulus sentence taken from our materials (the numbers refer to regions into which the stimulus was split for presentation, cf. Material subsection). (5b) and (5d) correspond to (1b) and (1d) in that they pair a telic directional phrase with a durational temporal adverbial in (5b) and a completive one in (5d). The telic directional phrase matches the completive adverbial in (5d) but mismatches the durative adverbial in (5b). We suggest that the aspectual conflict in (5b) is resolved by means of iterative coercion, as in (4); cf. Todorova et al. (2000). In (5a) and (5c), however, we replaced the atelic directional phrase *entlang der Küste* ‘along the coast’ from (1a) and (1c) with an underspecified directional phrase *über die Nordsee* ‘over the-acc North Sea’. The underspecified directional phrase is ambiguous, i.e. neutral relative to ±telic, and permits any coercion between the verb and the temporal adverbial to come to light. The *über*-phrase ‘over...’ in (5a) and (5c) allows for a telic interpretation in which the pirate moves along a straight path from one end of the North Sea to the other, together with a second, atelic, interpretation, in which he moves along an arbitrary path across the North Sea without reaching or passing its boarders (cf. Bierwisch 1988; Maienborn 1990).^3^ 
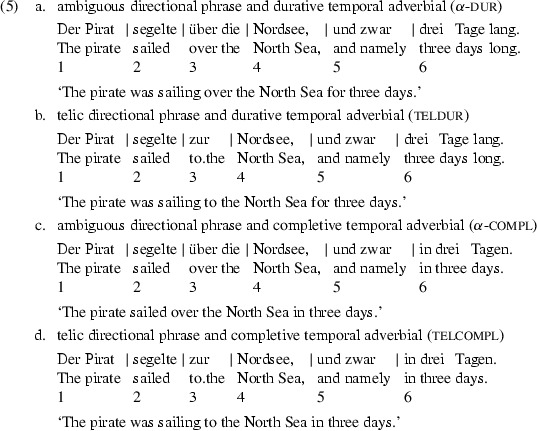


Unlike in (1), the temporal adverbials in (5) are put at the end of the sentences, the German *Nachfeld*, so that during processing the VP is built up before the temporal adverbial is attached. In this scenario, VPs with an ambiguous directional phrase are especially interesting because the Coercion Account and the Underspecification Account make different predictions with respect to their aspectual properties. On the Coercion Account, the VP carries the aspectual value of the verb, hence it is atelic; on the Underspecification Account, the VP remains aspectually underspecified because neither the verb nor the PP offer any aspectual specification.^4^

We derive differential predictions from the two rival accounts, Coercion versus Underspecification, for reading times on the directional phrase (region 4) and on the temporal adverbial (region 6), focusing in particular on the latter. Increased reading times in case of an aspectual conflict will be attributed to coercion as a conflict resolution mechanism.

During processing of the directional phrase, the complement is combined with the verb to yield the VP. Extra processing costs in VP construction should show up in increased reading times on the noun at the end of the directional phrase (region 4) or shortly afterwards (region 5). Building a VP from a motion verb and an ambiguous directional phrase *über die Nordsee* ‘over the North Sea’, as in (5a) and (5c), should be easy on both accounts. The resulting VP, however, differs in its aspectual property. According to the Coercion Account, the VP inherits the aspect from the verb and is specified as atelic. According to the Underspecification Account, the VP remains aspectually underspecified.

An aspectual conflict during VP construction arises whenever the aspect of the verb and of the directional phrase differ. This requires that the verb is aspectually specified as assumed by the Coercion Account only: motion verbs are lexically specified as atelic. An aspectual conflict arises when the atelic motion verb meets a telic directional phrase like *zur Nordsee* ‘to the North Sea’, as in (5b) and (5d). The conflict is resolved by verb coercion, leading to increased reading times on the directional phrase in conditions (5b) and (5d) compared to the conditions (5a) and (5c); the VP resulting in (5b) and (5d) is telic. The Underspecification Account also assumes that the VP in (5b) and (5d) will be telic. Verb coercion, however, is not required since the verb is lexically not specified with respect to aspect. The VP simply inherits the aspectual value from the directional phrase and reading times on region 4 should be about the same in all four conditions.

During processing of the temporal adverbial, the adverbial is combined with the VP. Aspectually sensitive temporal adverbials are used here as a means to induce a combinatorial conflict in case of a mismatch, as it has been shown that an aspectual mismatch between a temporal adverbial and a VP causes processing costs (Todorova et al. 2000; Seegmiller et al. 2004; Husband 2006). A durative adverbial like *drei Tage lang* ‘for three days’ in (5a) and (5b) matches an atelic VP but mismatches a telic one. In contrast, a completive adverbial like *in drei Tagen* ‘in three days’ matches a telic VP but mismatches an atelic one. Either kind of temporal adverbial matches an aspectually underspecified VP.

The Coercion Account and the Underspecification Account agree that the durative adverbial mismatches the VP in (5b) and matches the VP in (5a). Both accounts assume a mismatch in (5b), teldur, as they both consider the VP as telic; the agreement on the match in (5a), $\alpha$-dur, however, rests on different assumptions about the VP aspect. Whereas the Coercion Account assumes that the durational adverbial matches an atelic VP, the Underspecification Account assumes that the completive adverbial matches an underspecified VP. Due to this very difference in the representational assumptions for the VP, the two accounts generate different predictions for the completive adverbial. The Coercion Account assumes for condition (5c), $\alpha$-compl, that the completive adverbial mismatches an atelic VP, yet the Underspecification Account predicts that the completive adverbial matches an underspecified VP. The accounts again agree that the completive adverbial matches the telic VP in condition (5d), telcompl, and again for the same reason: the VP is telic.

To summarize the predictions for reading times on the critical region 6 (temporal adverbial), the Coercion Account expects two difficult conditions, teldur and $\alpha$-compl, and two easy conditions, $\alpha$-dur and telcompl; the Underspecification Account expects only one difficult condition, teldur, with the three other conditions being about equally easy.

In addition to the recorded reading times, acceptability judgements were prompted immediately after a sentence was read in the experiment. Acceptability is intended to reveal whether processing difficulties are reflected in this offline measure and thus provide a supplementary source of evidence for the evaluation of our predictions. The predictions correspond to the pattern predicted for reading times on the late region 6. The Underspecification Account predicts that the sentences in conditions teldur will be judged less acceptable than in the other three conditions. The Coercion Account expects that the sentences in teldur and $\alpha$-compl are judged less acceptable than in $\alpha$-dur and telcompl. If the difficulties in VP construction (region 4) affect acceptability, too, condition $\alpha$-dur will yield the highest acceptability as this is the only easy condition on the Coercion Account.

## Experiment 1: Self-Paced Reading Study

### Method

We implemented a self-paced reading study combined with an acceptability judgment task. The $2\times 2$-design was composed of the two within-factors directional phrase (telic vs. ambiguous) and temporal adverbial (completive vs. durative). The resulting four conditions are abbreviated as telcompl, teldur, $\alpha$-compl, $\alpha$-dur.

#### Participants

Forty-eight students from the University of Tübingen participated in the study and received a monetary reimbursement. They were all native speakers of German and were naïve to the purpose of the study.

#### Material

Materials consisted of 32 experimental sentences that were realized in all four conditions. A sample item is shown in (5); see the Appendix for a full list of experimental items. Experimental sentences were split into six regions corresponding to the arrangement in (5) indicated by the vertical lines. The regions of interest were the noun at the end of the directional phrase (region 4), the spill-over region 5 ‘and namely’, and the temporal adverbial (region 6).

Sixteen motion verbs were used: *fahren* ‘drive’, *fliegen* ‘fly’, *gehen* ‘go’, *klettern* ‘climb’, *kriechen* ‘crawl’, *laufen* ‘walk’, *marschieren* ‘march ’, *radeln* ‘cycle’, *rasen* ‘speed’, *reiten* ‘ride (a horse)’, *rennen* ‘run’, *schleichen* ‘sneak’, *schlendern* ‘amble’, *segeln* ‘sail’, *spazieren* ‘stroll’, *wandern* ‘hike’. Each verb was used in two experimental sentences.

The experimental sentences consisted of a subject (region 1), followed by a verb (region 2), then a directional phrase (regions 3 and 4) and finally a temporal adverbial (region 6). This order of constituents ensured that the aspect of the motion verb was not tainted by any preceding context that carries aspectual information. By using *und zwar* ‘namely’ (region 5), the temporal adverbial is moved to the German *Nachfeld* in order to provide for a syntactic configuration in which the adverbial is processed only after the VP has been built by combining the verb with the directional phrase. This sentence structure prevented us from including a sentence-final spill-over region because the resulting sentence would be visibly marked if not ungrammatical.

There were two levels of the factor directional phrase: (1) prepositional phrases that are ambiguous between a telic and an atelic reading, (5a) $\alpha$-dur and (5c) $\alpha$-compl; (2) prepositional phrases that are clearly telic (5b) teldur and (5d) telcompl. There were also two levels of the factor temporal adverbial: (1) durative adverbials as in (5a) $\alpha$-dur and (5b) teldur, match atelic VPs but mismatch telic ones; (2) completive adverbials as in (5c) $\alpha$-compl and (5d) telcompl, match telic VPs but mismatch atelic ones. Either kind of adverbial matches an aspectually underspecified VP.

In order to minimize the wording differences between the conditions, the internal arguments of telic and ambiguous directional phrases were the same [e.g. *Nordsee* ‘North Sea’ in (5)]. The internal arguments were chosen in such a way that the ambiguous directional phrase is unbiased relative to telicity. To mitigate any effects due to the difference in length of the prepositions, directional phrases were split into two regions, e.g. |*zur/über die*| and |*Nordsee*| with the second one, region 4, being the critical one for measuring reading times. Region 3 was not analyzed because the type of the prepositional phrase is not yet determined at this point. The sentence could be continued, e.g. in a directional, locative or temporal way (e.g. locative: *über der Nordsee* ‘over the-dat North Sea’; temporal: *über die letz-ten Wochen* ‘over the course of several weeks’ or *zur Mitternachtsstunde* ‘at the midnight hour’).

The experimental sentences were combined with 64 filler sentences, which were designed to resemble the experimental sentences. They consisted of an NP followed by a verb and a prepositional phrase (e.g. *der Betriebsleiter suchte nach einem Angestellten* ‘the manager was looking for an employee’). The second part of the filler sentences contained a conjunction and an adverbial (e.g. *aber nur mit wenig Enthusiasmus* ‘but with little enthusiasm’). In order to distract from the somewhat marked conjunction *und zwar* ‘and namely’ in the items, the fillers contained similar conjunctions (e.g. *und sogar* ‘and even’ and *aber doch* ‘but even so’). The conjunctions *und* ‘and’ and *aber* ‘but’ were counterbalanced across the whole set of 96 sentences ‘and’ occurred in all 32 experimental sentences and in 16 filler sentences.

The well-formedness of the filler sentences varied from completely acceptable to completely unacceptable. Ill-formed filler sentences contained grammatical violations (e.g. *Der Kanzler regierte während der Hauptstadt, aber doch aus reinem Egoismus.* ‘The Chancellor ruled during the capital, but out of pure selfishness.’) or world knowledge violations (e.g. *Die Reitschüler eilten über die Stalltür, aber ziemlich ungern.* ‘The riding students hurried over the stable door, but quite reluctantly.’). The mismatches occurred in the main sentence or in the continuation, mirroring the critical regions 4 and 6 of the experimental sentences.

The experimental sentences were distributed over four lists such that each of the four variants of a sentence was assigned to a different list and each list contained eight sentences in each of the four conditions. Experimental sentences were intermixed with filler sentences and randomized separately for each participant.

#### Procedure

The experiment was programmed using the E-Prime 2.0 Professional software (Psychology Software Tools, Inc.). Participants were tested individually at a PC. The main experiment was preceded by four practice trials. Sentences were presented to participants with a moving window technique. Presentation of regions was self-paced. After the first press of the space bar, the trial began with a display showing the whole stimulus sentence completely masked, i.e., all characters including spaces were substituted with dashes. With the second press of the space bar, region 1 was shown by replacing the dashes with the corresponding characters. Any further press of the space bar led to the remasking of the current region and a concurrent demasking of the subsequent region. After the presentation of a sentence was completed, a final press of the space bar revealed a prompt for an acceptability judgement on a five-point scale from 1 (*sehr schlecht* ‘very bad’) to 5 (*sehr gut* ‘very good’).

In order to encourage participants to read the sentences carefully, one third of the trials ended with a *yes*–*no* comprehension question, which was answered by means of two designated keys. Participants were instructed to read through the sentences at a natural pace and to read closely enough to be able to answer the questions. Experimental sessions lasted less than 30 min.

### Results

#### Reading Times

Reading times (RTs) were analyzed for regions 4–6. Outliers were excluded in two steps. Firstly, RTs shorter than 200 ms or longer than 5000 ms were discarded. Thereafter, RTs extending a participant’s mean on this region in the corresponding condition by more than 2.5 standard deviations were excluded. Overall, .6% of the data was removed from the analysis. The remaining reading times were submitted to repeated measures ANOVAs with participant ($F_{1}$) or item ($F_{2}$) as random factor.

Effects of directional phrase so as to indicate a conflict in VP construction were visible neither on region 4 (noun of directional phrase; 756 ms for ambiguous and 751 ms for telic directional phrases) nor on the spill-over region 5 (*und zwar* ‘namely’; 507 ms after ambiguous and 498 ms after telic directional phrases) [all $F\hbox {s}<1.1$]. This finding is consistent with the Underspecification Account and disagrees with the Coercion Account.

**Fig. 1:**
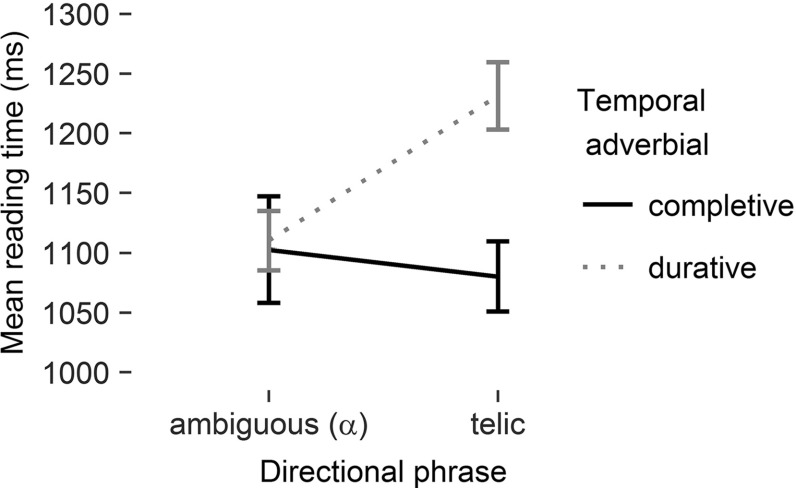
Mean reading times on region 6 as a function of directional phrase $\times$ temporal adverbial. *Error bars* correspond to standard errors of the mean

Mean reading times on the critical region 6, the sentence-final temporal adverbial, are presented in Fig. 1. The central outcome for region 6 is the significant interaction of the two factors, directional phrase and temporal adverbial [$F_{1}(1,47)=6.70$, $p<.05$, $\eta ^{2}=.13$; $F_2(1,31)=6.75$, $p<.05$, $\eta ^{2}=.18$]. Planned comparisons confirmed that it took longer to read the durative temporal adverbial in condition teldur compared to $\alpha$-dur [$t_{1}(47)=2.79$, $p<.01$; $t_{2}(31)=3.14$, $p<.01$], whereas it took the same amount of time to read the completive temporal adverbial in conditions telcompl and $\alpha$-compl [both $t\hbox {s}<1$]. The interaction pattern confirms the Underspecification Account and is not in line with the Coercion Account.

The main effects of temporal adverbial [$F_{1}(1,47)=3.86$, $p=.06$, $\eta ^2=.08$; $F_{2}(1,31)=5.52$, $p<.03$, $\eta ^2=.15$] and directional phrase [$F_{1}(1,47)=3.03$, $p=.09$, $\eta ^2=.06$; $F_{2}(1,31)=1.65$, $p>.10$] did not consistently reach significance.

**Fig. 2:**
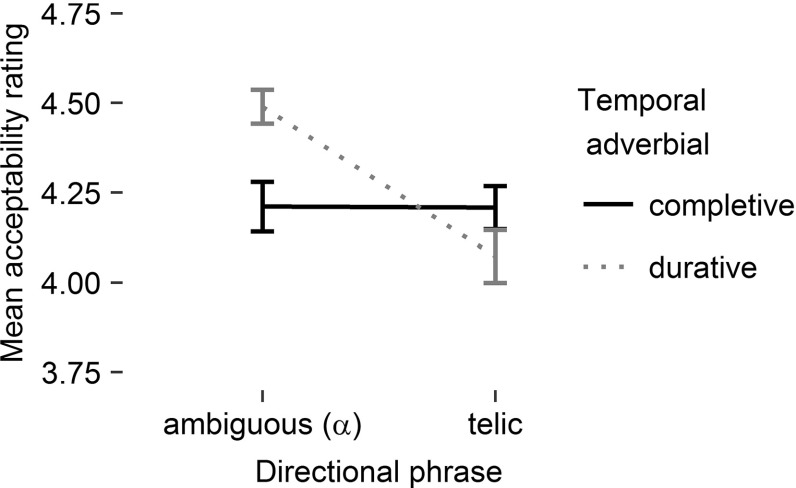
Mean acceptability ratings (1 “very bad” to 5 “very good”) as a function of directional phrase $\times$ temporal adverbial. *Error bars* correspond to standard errors of the mean

#### Acceptability Judgements

Mean acceptability judgements (1 $=$ ‘very bad’ to 5 $=$ ‘very good’) are summarized in Fig. 2. Most importantly, the interaction of temporal adverbial with directional phrase again turned out to be significant [$F_{1}(1,47)=25.51$, $p<.001$, $\eta ^2=.35$; $F_{2}(1,31)=13.33$, $p<.001$, $\eta ^2=.30$]. directional phrase had an effect if the temporal adverbial was durative but not if it was completive. Acceptability is significantly lower in teldur compared to $\alpha$-dur [$t_{1}(47)=5.91$, $p<.001$; $t_{2}(31)=4.89$, $p<.001$], whereas acceptability does not differ between telcompl and $\alpha$-compl [both $t\hbox {s} < 1.2$]. As with reading times, the interaction pattern for acceptability corroborates the Underspecification Account and goes against the Coercion Account.

In this analysis, the interaction goes along with a main effect of directional phrase [$F_{1}(1,47)=19.04$, $p<.001$, $\eta ^2=.29$; $F_{2}(1,31)=13.44$, $p<.001$, $\eta ^2=.30$] but without a main effect of temporal adverbial. The main effect is primarily a side-effect of the two-way interaction.

### Discussion

The results of the experiment confirm the predictions of the Underspecification Account and speak against the Coercion Account. The interaction pattern of the two factors, directional phrase and temporal adverbial, found in reading times on region 6 as well as in acceptability is due to a single difficult condition, teldur, instead of two difficult conditions, teldur and $\alpha$-compl.

The results indicate that the combination of a standard motion verb and an ambiguous directional phrase results in an underspecified verbal phrase. In this case, the aspectual specification of the VP doesn’t take place earlier than its combination with the temporal adverbial. This is supported by the absence of any processing difficulties in either $\alpha$-condition, i.e., regardless of the kind of temporal adverbial the VP is combined with. In the two tel-conditions, the VP is specified to a telic interpretation before encountering the temporal adverbial. This telic VP conflicts with a following mismatching durative adverbial in the teldur condition. Here, the temporal adverbial takes longer to read than in the $\alpha$-dur condition. Convergent evidence is provided by the missing increase in reading times on the directional phrase (region 4), which would be expected if a lexically atelic motion verb combines with a telic directional phrase.

We will now address a possible explanation why our experiment did not support the Coercion Account’s hypothesis although the aspectual value of motion verbs is in fact lexically specified. This possibility rests on the idea that motion verbs are lexically specified without being consistently specified as atelic. Instead, some motion verbs are specified as atelic, whereas other motion verbs are specified as telic.^5^ Such a distinction should come along with a more frequent use of a verb with the lexically specified reading.

If the sample of motion verbs used in our experiment turns out to consist of a mixture of atelic and telic motion verbs, a combination with an ambiguous directional phrase would result in a corresponding mixture of atelic and telic VPs. Afterwards, when encountering a temporal adverbial, atelic and telic VPs behave oppositely. Atelic VPs match durative adverbials but mismatch completive adverbials (predicted by the Coercion Account); telic VPs match completive adverbials but mismatch durative adverbials (analogous to teldur). Processing difficulties are therefore to be expected to sometimes occur in either condition with an ambiguous directional phrase, in $\alpha$-dur with a telic verb and in $\alpha$-compl with an atelic verb. Averaged across all verbs, the two $\alpha$-conditions may then yield similar results. If we succeed in teasing telic verbs apart from atelic ones, a re-analysis of the present data should reveal the opposite behavior of telic and atelic motion verbs.

This alternative explanation is problematic for the Coercion Account, because it presupposes that not all motion verbs are atelic. In the following part of the paper, we explore the adjusted predictions of the original Coercion Account, henceforth called the Modified Coercion Account. These predictions are contrasted with those of the Underspecification Account in two verb bias experiments: a corpus study and a completion study.

## Verb Bias Experiments

In order to examine whether our sample of motion verbs consists of atelic and telic verbs that yield opposite effects when combined with an ambiguous directional phrase, we have to determine the presumed telicity of the verbs. We assume that activities are preferably expressed by atelic verbs whereas accomplishments are preferably expressed by telic verbs. Hence, lexically atelic verbs should be used more frequently in descriptions of activities (atelic verb occurrences) and lexically telic verbs should be used more often in descriptions of accomplishments (telic verb occurrences).

We identified the telicity bias of the 16 motion verbs (atelic vs. telic) by determining the relative frequency of atelic and telic occurrences in two ways: a corpus study and a completion study with a sentence completion task. Under the assumption that some of the standard motion verbs are lexically specified as atelic, whereas others are lexically specified as telic, we would expect to find a high congruency in the telicity biases for our motion verbs in the two studies. Furthermore, we would expect a bimodal frequency distribution of motion verbs as a function of the telicity bias. That means that few—if any—verbs show about an equal number of atelic and telic occurrences (i.e., no telicity bias); accordingly, there should be two maxima, one below that minimum (atelic bias) and one above it (telic bias).^6^

### Corpus Study

#### Method

200 occurrences of each experimental verb were extracted from the *Deutsches Referenzkorpus* using Cosmas II (CoSMAS 2008; Kupietz and Keibel 2009). Every occurrence was accompanied by the last two sentences of the preceding context. Only examples containing finite forms of intransitive, literal uses of verbs were chosen for the analysis. Two annotators classified the verbs’ occurrences as “telic”, “atelic” or “ambiguous”, based on standard diagnostics for (a)telicity (see, e.g. Dowty 1979, p. 60). Ambiguous cases were discarded; occurrences were labeled “ambiguous” if the two annotators classified them oppositely or agreed on an ambiguous interpretation. Example (6) is taken from the corpus and demonstrates a telic and an ambiguous occurrence of two verbs, ‘run’ and ‘walk’, within a single sentence. ‘Run’ was classified as telic by both annotators and ‘walk’, was classified as ambiguous by both annotators and was therefore discarded. The annotation procedure led to a corpus of 2392 occurrences (113–184 per verb) of which 1470 (61%) were telic ones. 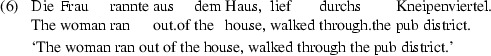


#### Results and Discussion

**Fig. 3:**
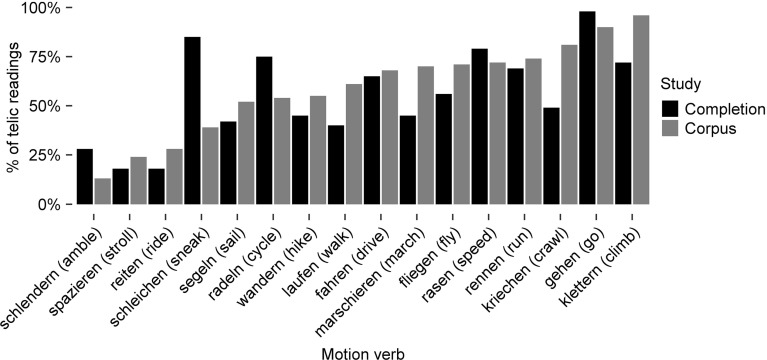
The distribution of telic readings of motion verbs determined by the corpus and the completion data. The verbs are ordered from *left* to *right* with increasing relative frequencies of telic readings in the corpus study

The results are displayed in Fig. 3. Telic verb interpretations ranged from very infrequent (13%: *schlendern* ‘amble’) to very frequent (96%: *klettern* ‘climb’). Such a broad range is consistent with the idea that some motion verbs are lexically specified as atelic and exhibit an atelic bias whereas other motion verbs are specified as telic and exhibit a telic bias. However, there is no indication of a bimodal frequency distribution of the verbs as a function of the telicity bias. Indeed there are verbs which do not exhibit much of a telicity bias, i.e., atelic and telic occurrences appear about equally often. Rather then a point of discontinuity at 50%, our sample of motion verbs shows a smoothly increasing telicity bias. The distribution bears resemblance to what Bott (2010, p. 117) reports of corpus data on a broader class of verbs.

### Completion Study

#### Method

For the paper-and-pencil sentence completion study, the 32 experimental sentences from the reading time experiment were used. To prompt completions, the sentences were truncated after the verb [see (7)]. Note that the sentence fragments did not differ between the four conditions of the reading time experiment. The stimuli were presented in booklets in one of four pseudo-randomized orders. The questionnaire began with three examples of continuations. The examples did not contain motion verbs and featured three kinds of prepositional phrases: a locative, a directional telic and a directional ambiguous one.

Thirty-two students of the University of Tübingen, all native speakers of German, participated in the study as part of introductory courses in linguistics. They were asked to complete the sentence fragments with locative or directional information that seemed natural to them. 
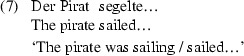


The two annotators from the corpus study classified the 1024 completions following the same criteria as in the corpus study. Completions were discarded if they contained adverbials not belonging to the required locative or directional type, particles, absentives, ambiguous completions and completions resulting in a different argument structure. Examples for included and excluded completions are presented in (8a) and (8b), respectively. A total number of 659 completions (29–61 per verb) remained for the analysis, of which 385 (58%) were telic ones. 
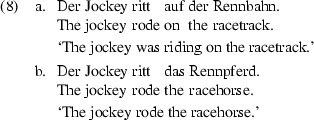


Each annotator classified half of the completions. 200 randomly chosen items per annotator were cross-checked by the other annotator, yielding 90% agreement. Additionally, two further random samples of 200 items per annotator were cross-checked by two further annotators, yielding 91% agreement.

#### Results and Discussion

The results are displayed in Fig. 3. Telic verb occurrences range from infrequent (18%: *schlendern* ‘amble’) to predominant (98%: *gehen* ‘go’). As in the corpus study, we observed a continuous increase in the telicity bias instead of a bimodal distribution. The two studies agree that about 60% of all occurrences are telic ones, that is, a general lexical specification of our motion verbs as atelic would require coercion in more than half of all observed occurrences.

The proportions of telic readings for the verbs were similar to that found in the corpus study, as confirmed by Spearman’s rank correlation [$\rho =.60$, $t(14)=2.81$, $p<.05$]. Obviously, some motion verbs frequently occur with an atelic reading, while others frequently occur with a telic reading.

In sum, there is no evidence for a bimodal frequency distribution in either of the verb bias studies. Instead there is a broad spectrum of aspectual values. In order to thoroughly explore the hypothesis derived from the Modified Coercion Account, we nevertheless distinguish between verbs with more and with less frequent telic occurrences.

### Teasing ‘Atelic’ and ‘Telic’ Motion Verbs Apart

Based on the telicity bias results of the corpus and the completion study, we subdivided our sample of 16 motion verbs into ‘atelic’ and ‘telic’ verbs (cf. Footnote 6). We did this by first applying a split-half division to either data set: verbs at ranks 1–8 were preliminarily classified as telic, verbs at ranks 9–16 as atelic. Four verbs fell into different halves in the two data sets: *kriechen* ‘crawl’ at ranks 3 and 9, *marschieren* ‘march’ at ranks 7 and 9, *radeln* ‘cycle’ at ranks 11 and 4, and *schleichen* ‘sneak’ at ranks 13 and 2 of the corpus and the completion data set, respectively. These four verbs were discarded in order to obtain a clearer distinction between the two verb groups. With the removal of the four verbs, Spearman’s $\rho$ increased to .81, which is significant with $t(10)=4.37$. The remaining 12 verbs were separated into six ‘atelic’ verbs (mean frequency of telic readings 35%, *schlendern* ‘amble’, *spazieren* ‘stroll’, *reiten* ‘ride (a horse)’, *segeln* ‘sail’, *wandern* ‘hike’, *laufen* ‘walk’) and six ‘telic’ verbs (mean 76%, *fahren* ‘drive’, *fliegen* ‘fly’, *rennen* ‘run’, *rasen* ‘speed’, *klettern* ‘climb’, *gehen* ‘go’). Based on this division, we proceeded to re-analyze the data from the self-paced-reading experiment.

#### Re-analyses of Reading Times and Acceptability Judgements

The verification of the Modified Coercion Account is most straightforward if the re-analyses of reading times on region 6 and acceptability judgements are restricted to ambiguous directional phrases. Combining a lexically specified verb with an ambiguous directional phrase results in an atelic VP if the verb is specified as atelic and in a telic VP if the verb is telic. Since an atelic VP mismatches a completive adverbial and a telic VP mismatches a durative adverbial, the Modified Coercion Account predicts higher reading times and lower acceptability judgements in condition $\alpha$-compl compared to $\alpha$-dur for ‘atelic’ verbs, but the opposite for ‘telic’ verbs. Consequently, the Modified Coercion Account predicts an interaction between temporal adverbial (‘durative’ vs. ‘completive’) and telicity bias (‘atelic’ vs. ‘telic’).

It turned out that ‘atelic’ and ‘telic’ verbs were distributed across the four lists in a way that the two kinds of verbs did not always occur in all four conditions. We therefore performed only $F_{2}$-analyses with item as random factor. The $F_{2}$-analyses are based on 24 items (12 verbs) with temporal adverbial as within factor and telicity bias as between factor.

The analysis of the RTs on region 6 yielded a main effect of verb bias [$F_{2}(1,22)=4.99$, $p<.05$, $\eta ^{2}=.19$], but not of temporal adverbial [$F_{2}(1,22)=2.87$, $p=.10$, $\eta ^{2}=.12$]. The interaction was not significant [$F_{2}(1,22)=2.81$, $p=.11$, $\eta ^{2}=.11$]. Since the worst condition (longest RTs) is the ‘telic’ verb in combination with a completive temporal adverbial (cf. Table 1), the pattern goes against the predictions of the Modified Coercion Account, anyway.

A similar pattern is observed in the acceptability judgements. The main effects of verb bias [$F_{2}(1,22)=7.92$, $p=.01$, $\eta ^{2}=.27$] and temporal adverbial [$F_{2}(1,22)=8.22$, $p<.01$, $\eta ^{2}=.27$] are both significant; the interaction of the two factors is marginal [$F_{2}(1,22)=3.65$, $p=.07$, $\eta ^{2}=.14$]. As evident from Table 1 and contrary to the prediction of the Modified Coercion Account, sentences with telic verbs were rated low when combined with a completive temporal adverbial; there is no difference between the atelic verbs. The low acceptability of ‘telic’ verbs combined with a completive adverbial corresponds to the high reading times on region 6.^7^

**Table 1:** Mean RTs (in ms) on region 6 and mean acceptability ratings for ambiguous directional phrases in the re-analysis of the reading-time experiment

Verb bias	Temporal adverbial	Reading times on reg. 6		Acceptability ratings	
		Mean	SE	Mean	SE
Atelic	Durative	1056	44	4.56	.08
	Completive	1057	68	4.47	.10
Telic	Durative	1105	44	4.45	.08
	Completive	1282	68	4.03	.10

### Discussion of the Re-analyses

To summarize, the verb bias studies and, in particular, the re-analyses of reading times on region 6 and acceptability did not provide any evidence in support of the Modified Coercion Account. The Modified Coercion Account assumes that motion verbs are lexically specified as −telic for some of the verbs and $+$telic for the other verbs. The modification did not help the Coercion Account to cope with the findings. We therefore generalize the conclusion that the findings run counter the Coercion Account, with and without modification. We maintain our initial interpretation of the results: reading times and acceptability judgements provide convergent evidence in favor of the Underspecification Account and in disagreement with the Coercion Account. Since the telicity bias of motion verbs turned out to be irrelevant for aspectual meaning constitution, the bias, as observed in rather close correspondence in the corpus and in the sentence completions, cannot be attributed to a lexical specification of aspect. The differences in the relative frequencies of atelic and telic occurrences of the verbs in the corpus and the completion study did not induce a systematic difference in the semantic composition of the VPs.

## General Discussion

Coercion and underspecification are mechanisms of meaning adaption which correspond to empirically observable phenomena. During processing, the resolution of a combinatory conflict is tied with increased cost, while the specification of an underspecified structure proceeds effortlessly. The question addressed here was whether the distinction between coercion and underspecification can be made experimentally. Motion verbs are a suitable subject of investigation for comparing these mechanisms, because theories based on coercion and underspecification make clear and opposing predictions for the processing of motion verbs.

We reported one experiment, flanked by two complementing studies, that addressed the question whether typical motion verbs are aspectually underspecified or have a lexically specified atelic meaning, i.e. can be classified as activity verbs. The evidence included reading times and acceptability judgements from the main experiment, as well as corpus and completion data.

Both the Coercion Account and the Underspecification Account expected an aspectual mismatch of a durative adverbial with a preceding telic directional phrase (teldur) but not with an ambiguous one ($\alpha$-dur). The ambiguous directional phrase results in an underspecified VP (Underspecification Account) or in an atelic VP (Coercion Account), neither of which conflicts with the durative adverbial. The higher reading times on region 6 and the decrease in acceptability confirm this expectation, in line with Todorova et al. (2000) and Piñango et al. (1999): Where necessary, aspectual coercion takes place and is associated with processing costs.

The Coercion Account predicted an additional conflict between the ambiguous VP and the completive temporal adverbial ($\alpha$-compl), where the VP is coerced to a telic interpretation. In contrast, the Underspecification Account predicted that the specification of the ambiguous VP to a telic reading when combined with the sentence final temporal adverbial should proceed as effortlessly as the integration of the completive temporal adverbial into the telic VP (telcompl). No processing differences were observed between the $\alpha$-compl and telcompl conditions. The combination of a standard motion verb with the ambiguous directional phrase yields an underspecified VP, which receives its aspectual assignment only in combination with the temporal adverbial. This finding provides evidence against the Coercion Account and in favour of the Underspecification Account.

We explored the Modified Coercion Account as an explanation for the absence of the differences between $\alpha$-compl and telcompl predicted by the original Coercion Account. If standard motion verbs are not an aspectually homogeneous group but in fact divide into two subgroups with respect to their lexically specified aspect, then the Coercion Account’s original hypothesis applies only to atelic verbs. The predicted effect could have been blurred by telic verbs in the sample. Two control studies aimed at exploring this possibility. The Modified Coercion Account predicted that some motion verbs are telic and others are atelic. Given this distinction, a VP resulting from a combination of a lexically specified verb with an ambiguous directional phrase inherits the verb aspect. The processing difficulty claimed by the Coercion Account’s original hypothesis for any motion verb would then be restricted to atelic verbs (conflict in $\alpha$-comp), whereas the opposite holds for telic verbs (conflict in $\alpha$-dur).

The re-analyses did not confirm that the telicity bias, obtained from the corpus and completion data, has a lexical basis. Combined frequencies from the two verb bias studies were used to separate presumably atelic from presumably telic verbs. The re-analysis of the reading times and acceptability data with 12 verbs did not confirm the predicted effects of verb telicity. This result speaks against the Modified Coercion Account and strengthens the argument in favor of the Underspecification Account. The Underspecification Account postulates a standard compositional integration of the prepositional phrases into the meaning of the verbal projection, and can therefore best explain the observed effects. The studies showed that the observed telicity inclination of verbs does not play a visible role in the compositional process. Further work is needed in order to fully explain consistent telicity effects found in the control studies and why these preferences showed no corresponding effect on processing.

In studying the combination of motion verbs with directional phrases, the present work investigated the phenomena of underspecification and coercion in a new empirical domain. Furthermore, we combined this new empirical domain with the well-known case of temporal adverbials, in order to obtain a reliable diagnostics for aspectual coercion. This design proved valuable in contributing new experimental evidence to the theoretical dispute on whether or not standard motion verbs are aspectually specified in the mental lexicon.

To conclude, the work presented in this paper contributes experimental evidence, supplemented by corpus evidence, to the theoretical discussion of the mechanisms of meaning variability. Whether a certain phenomenon of flexible meaning beyond a purely compositional computation of meaning is an instance of underspecification or coercion is not a mere matter of theoretical taste but can be decided upon experimentally. We observed the undisputed coercion effect (in teldur) but we did not observe the disputed coercion effects (in $\alpha$-conditions on region 5 and in $\alpha$-compl on region 6), and hence rejected both the original and the Modified Coercion Account. Therefore, our study provides experimental confirmation for the use of both mechanisms, underspecification and coercion, as means to achieve sufficient flexibility in natural language interpretation. Furthermore, the study fits in well with the available evidence that coercion, i.e., the resolution of a combinatory conflict, causes additional processing costs, whereas the specification of an underspecified, yet grammatically well-formed structure, proceeds without additional effort. These findings provide significant insights for linguistic theory, since they indicate that the theoretical modeling of meaning variability cannot be reduced to one single mechanism, underspecification or coercion, but requires both. An experimental approach as the one used here suggests that the stipulation of a combinatory conflict should be independently attested. Thus, there are theoretical and experimental reasons to raise the bar for considering underspecification and coercion in the theoretical modeling of meaning variability.

## Appendix: Materials of the Reading Time Experiment

1. Der Lehrer ging {über den / zum} Schulhof, und zwar {zwei Minuten lang. / in zwei Minuten.}
2. Der Ingenieur ging {über die / zur} Großbaustelle, und zwar {zwanzig Minuten lang. / in zwanzig Minuten.}
3. Der Mann spazierte {über den / zum} Bahnhofsvorplatz, und zwar {zehn Minuten lang. / in zehn Minuten.}
4. Der Rentner spazierte {über den / zum} Friedhof, und zwar {zehn Minuten lang. / in zehn Minuten.}
5. Die Spinne kletterte {über die / zur} Kommode, und zwar {dreißig Sekunden lang. / in dreißig Sekunden.}
6. Der Dachdecker kletterte {über das / zum} Gerüst, und zwar {zehn Minuten lang. / in zehn Minuten.}
7. Der Student wanderte {über die / zur} Bergkette, und zwar {zwei Wochen lang. / in zwei Wochen.}
8. Der Tourist wanderte {über die / zur} Hochebene, und zwar {zwei Stunden lang. / in zwei Stunden.}
9. Der Fahranfänger raste {über den / zum} Marktplatz, und zwar {vierzig Sekunden lang. / in vierzig Sekunden.}
10. Der Rennfahrer raste {über die / zur} Rennstrecke, und zwar {sieben Minuten lang. / in sieben Minuten.}
11. Die Hausfrau schlenderte {über den / zum} Wochenmarkt, und zwar {zwanzig Minuten lang. / in zwanzig Minuten.}
12. Der Wanderer schlenderte {über die / zur} Lichtung, und zwar {zehn Minuten lang. / in zehn Minuten.}
13. Der Trainer lief {über den / zum} Sportplatz, und zwar {zwanzig Sekunden lang. / in zwanzig Sekunden.}
14. Der Polizist lief {über den / zum} Parkplatz, und zwar {acht Minuten lang. / in acht Minuten.}
15. Die Maus rannte {über das / zum} Grundstück, und zwar {zwanzig Sekunden lang. / in zwanzig Sekunden.}
16. Die Kinder rannten {über den / zum} Spielplatz, und zwar {drei Minuten lang. / in drei Minuten.}
17. Das Modellflugzeug flog {über die / zur} Zuschauertribüne, und zwar {eine Minute lang. / in einer Minute.}
18. Der Pilot flog {über das / zum} Kriegsgebiet, und zwar {vier Stunden lang. / in vier Stunden.}
19. Der Pirat segelte {über die / zur} Nordsee, und zwar {drei Tage lang. / in drei Tagen.}
20. Die Crew segelte {über das / zum} Mittelmeer, und zwar {fünf Tage lang. / in fünf Tagen.}
21. Der Kopilot fuhr {über das / zum} Rollfeld, und zwar {zehn Minuten lang. / in zehn Minuten.}
22. Der Panzer fuhr {über das / zum} Schlachtfeld, und zwar {fünf Stunden lang. / in fünf Stunden.}
23. Das Mädchen ritt {über das / zum} Stoppelfeld, und zwar {fünfzehn Minuten lang. / in fünfzehn Minuten.}
24. Der Jockey ritt {über die / zur} Weide, und zwar {drei Minuten lang. / in drei Minuten.}
25. Der Offizier marschierte {über den / zum} Kasernenplatz, und zwar {sieben Minuten lang. / in sieben Minuten.}
26. Die Pfadfinder marschierten {über den / zum} Zeltplatz, und zwar {fünfzehn Minuten lang. / in fünfzehn Minuten.}
27. Der Ehemann schlich {über den / zum} Hinterhof, und zwar {zwei Minuten lang. / in zwei Minuten.}
28. Die Katze schlich {über die / zur} Terrasse, und zwar {dreißig Sekunden lang. / in dreißig Sekunden.}
29. Die Schnecke kroch {über das / zum} Blumenbeet, und zwar {zwanzig Minuten lang. / in zwanzig Minuten.}
30. Der Schauspieler kroch {über die / zur} Bühne, und zwar {vier Minuten lang. / in vier Minuten.}
31. Die Kinder radelten {über die / zur} Schotterpiste, und zwar {zwei Stunden lang. / in zwei Stunden.}
32. Der Mitarbeiter radelte {über das / zum} Gelände, und zwar {eine Stunde lang. / in einer Stunde.}

## Footnotes

1 Throughout the paper, we use the term “aspect” to refer to the temporal constituency of the eventualities denoted by the expressions; cf. Smith’s (1991) notion of “situation aspect” (often also called “lexical aspect”) as opposed to “viewpoint aspect” or “grammatical aspect”; see, e.g. Rothstein (2004). Note, furthermore, that the aspectual opposition [±telic] applies to eventualities but strictly speaking not to the paths denoted by directional phrases. The latter can be classified as [±bounded]. Yet, for the sake of simplicity we use the feature [±telic] for temporal and spatial (un)boundedness, i.e. for both eventualities and paths.

2 The underspecification formalisms developed, e.g. by Dölling (2003, 2014) and Egg (2005) are so powerful that they would also analyze *Sue began the book.* as an instance of underspecification, which is generally taken to be *the* paradigm case for coercion. Asher (2011), in contrast, proposes a formalism that is rather coercion-biased. For example, Asher (2011, pp. 15, 248) takes the ambiguity of *The dress with the flowers.* to involve coercion. Besides a fully compositional (yet dispreferred) reading, where a dress is arranged together with some real flowers, he assumes that the preferred reading, according to which some representation of flowers is drawn or stitched on the fabric of the dress, is based on coercion.

3 Note that the *über*-PP in (5) is overtly marked with accusative case and therefore unequivocally a directional phrase. That is, there is no danger of confounding directional PPs with locative PPs in German, because the latter are marked with dative case; e.g. *über der Nordsee* ‘over the-dat North Sea’.

4 We argue that there is no reason to assume that the VP is consistently specified as telic or atelic, if an underspecified verb is combined with an unbiased directional phrase; cf. Swets et al. (2008).

5 To our knowledge, there is only one short reference to an account of motion verbs as lexically specified to +telic, i.e. accomplishments in Rapp (1997, p. 110 f).

6 For the sake of brevity, we will speak of ‘atelic’ verbs and ‘telic’ verbs when the corresponding reading in the following studies is the predominant one.

7 We thank Lyn Frazier for suggesting to us correlation analyses as an alternative. For each item (N $=$ 32) we computed the differences of the means between the conditions with ambiguous directional phrases ($\alpha$-dur and $\alpha$-compl), both for reading times on region 6 and for acceptability judgments. We ran correlation analyses with the log-transformed frequencies of the verbs obtained in the completion study on the one hand, and the corpus study on the other. Consistent with the presented analyses, frequencies correlated with differences neither in reading times (*rs* smaller than .10 with *ps* not less than .65), nor in acceptability (*rs* smaller than .15, *ps* not less than .40).
